# The Efficacy of Soleus Push-Up in Individuals with Prediabetes: A Pilot Study

**DOI:** 10.3390/sports13030081

**Published:** 2025-03-10

**Authors:** Dávid Elek, Miklós Tóth, Balázs Sonkodi, Pongrác Ács, Gábor L. Kovács, Péter Tardi, Csaba Melczer

**Affiliations:** 1 Musculoskeletal Rehabilitation Department, Fejér County Szent György University Teaching Hospital Csákvár, 8083 Csákvár, Hungary; 2 Faculty of Health Sciences, Doctoral School of Health Sciences, University of Pécs, 7624 Pécs, Hungary; 3 Faculty of Health Sciences, Institute of Physiotherapy and Sport Science, University of Pécs, 7624 Pécs, Hungary; 4 Physical Activity Research Group, Szentágothai Research Centre, University of Pécs, 7624 Pécs, Hungary; 5 Department of Health and Sports Medicine, Hungarian University of Sports Sciences, 1123 Budapest, Hungary; 6 Institute of Laboratory Medicine, Semmelweis University, 1089 Budapest, Hungary; 7 Department of Sports Medicine, Semmelweis University, 1122 Budapest, Hungary; 8 National Laboratory on Human Reproduction, University of Pécs, 7624 Pécs, Hungary; 9 Molecular Medicine Research Group, Szentágothai Research Centre, University of Pécs, 7624 Pécs, Hungary; 10 Faculty of Health Sciences, Complex Sport Performance Diagnostic and Physiotherapy Research Institute, University of Pécs, 7624 Pécs, Hungary

**Keywords:** soleus push-up, SPU, blood glucose levels, prediabetic condition, metabolism

## Abstract

Background/Objectives: Hamilton and colleagues invented the soleus push-up exercise and showed that this exercise method was successful in reducing postprandial blood glucose levels in sedentary individuals. The objective of the current pilot study was to assess the efficacy of the soleus push-up in individuals with prediabetes and to evaluate the feasibility of incorporating this exercise method into their daily routine. Methods: Ten participants (mean age: 53.3 ± 2.7 years; four females, six males) with prediabetes were included in the study. Initially, participants underwent an oral glucose tolerance test (OGTT) while being sedentary to establish baseline postprandial blood glucose measurements. During a subsequent OGTT, participants concurrently performed the soleus push-up (SPU) exercise either with or without electromyographic (EMG) feedback. Blood glucose levels were measured at 15 min intervals over the two-hour duration of both OGTTs. Results: We observed that performing the SPU in a sitting position during the oral glucose tolerance test resulted in approximately a 32% reduction in postprandial glucose excursion compared to the sedentary baseline results. This effect was also present in the absence of EMG feedback. Conclusions: Our findings suggest that this repetitive, prolonged contractile muscle activity can improve metabolic regulation in prediabetic individuals without the need for a laboratory setting. SPU may be a viable and effective exercise to support metabolic health in home or work environments. However, further validation is needed with a larger sample size.

## 1. Introduction

It is becoming increasingly evident that the major technological advances of recent decades, through their impact on the social fabric and social connections, are contributing to a downward trend in the physical activity of the population in developed countries. In 2016, it was observed that 28% of the world’s adult population and 81% of adolescents were below the WHO guidelines for health promotion, which recommend 150 min of moderate-intensity physical activity per week for adults, or 75 min of high-intensity physical activity per week for adults, and 60 min of moderate-intensity physical activity per day for adolescents [1].

It is worth noting that a caloric surplus, which can result from a combination of reduced physical activity and a calorie-dense diet, may contribute to the development of obesity and a high BMI. It is estimated that more than 10% of the world’s population is overweight, which is known to be a significant risk factor for several diseases, including cardiovascular disease, orthopedic conditions, and type 2 diabetes mellitus. Hungary is placed fourth in the ranking of countries with overweight in the adult population among those in the Organization for Economic Co-operation and Development (OECD). It would be fair to say that a significant proportion of the adult population in the country is overweight, which is a factor that can contribute to a range of health issues, including cardiovascular disease, cancer, and metabolic disorders such as type 2 diabetes [2].

It is estimated that in Hungary, in addition to 110,000 undiagnosed cases of type 2 diabetes, around 661,000 people have diabetes, which represents a prevalence of 9.1%. It is also worth noting that 237,000 people in Hungary have impaired glucose tolerance (IGT) and 306,000 people have elevated fasting blood glucose (IFG). IGT and IFG are precursors to type 2 diabetes [2]. In 2019, the social security system allocated a total of HUF 8 billion for the provision of specialized care for patients with diabetes mellitus. Of this amount, 70%, or HUF 5.8 billion, was allocated to the funding of type 2 diabetes care [3,4].

Many studies have sought to treat prediabetes, resulting from the lifestyle attitudes mentioned above, with varying degrees of success. However, a research study published in 2022 at the University of Houston found that repeating a previously unstudied exercise method called the “Soleus Push up” (SPU) for three hours resulted in a notable improvement in VLDL and triglyceride levels, as well as glucose tolerance in the participants [5]. It seems that this form of exercise may have induced a large systemic effect through the isolated contraction of the soleus muscle, which has a predominantly slow oxidative fiber composition and accounts for ~1% of total body mass. In addition, it appears that it may have enhanced local oxidative metabolism. Given that the movement can be performed in a sitting position for extended periods without fatigue, it has the potential to be a convenient yet effective method of metabolic enhancement that could be incorporated into daily activities. Upon reviewing the demographic data of the participants in the trial, it became evident that there was a considerable range in the observed parameters. Nevertheless, the findings indicated that SPU was effective in improving glucose regulation at low physical activity levels [5].

The aim of our pilot study was to investigate the potential benefits of SPU for people with prediabetes and to analyze its clinical applications.

## 2. Materials and Methods

### 2.1. Participants

This study targeted individuals with prediabetes, defined by the World Health Organization (WHO) criteria as fasting blood glucose levels between 6.1 and 6.9 mmol/L and 120 min post-oral tolerance test (OGTT) glucose levels below 11.1 mmol/L [6,7]. Participants were recruited from the Csákvár sampling site at the SYNLAB Hungary Limited Liability Company Institute, where individuals who underwent glucose load testing, indicated by OENO codes 21310 and 23130, were eligible to apply. Of the 13 individuals who expressed interest, 3 were excluded due to failure to attend scheduled laboratory visits, while the remaining 10 participants (mean age: 53.3 ± 2.7 years; 4 females, 6 males) met the inclusion criteria. Exclusion criteria included fasting blood glucose levels exceeding 7 mmol/L, 120 min OGTT levels ≥11.1 mmol/L, use of glucose tolerance medication, or failure to attend any scheduled measurements.

Following the provision of initial verbal and subsequent written information, the participant was able to formalize their intention to participate by completing a consent form in accordance with the requirements set out in Decree 23/2002 (9 May 2002), issued by the Ministry of Health, and in compliance with the World Medical Association Declaration of Helsinki.

### 2.2. Study Design

All 13 applicants initially underwent a two-hour OGTT while remaining sedentary, which served as the baseline measurement. Blood glucose levels were recorded every 15 min. Approximately one week later, participants attended an SPU education session. Two applicants failed to attend, reducing the number of participants to 11. These participants were then randomly assigned to either the “EMG_SPU group” (*n* = 5) or the ”SPU group” (*n* = 6) using a software program (Microsoft Corporation, Redmond, WA, USA, 2402 build version 16.0.17328.20124 64-bit). In the following days, the second OGTT was conducted. During this test, participants performed SPU with (“EMG SPU group”) or without (“SPU group”) EMG feedback, while blood glucose levels were again measured every 15 min. One additional participant failed to attend, leaving 10 participants for the final analysis. Each participant completed both a baseline and an intervention OGTT, serving as their own control. The blood glucose results from the second OGTT were compared within and between groups to assess the effects of the SPU intervention.

**Figure 1:**
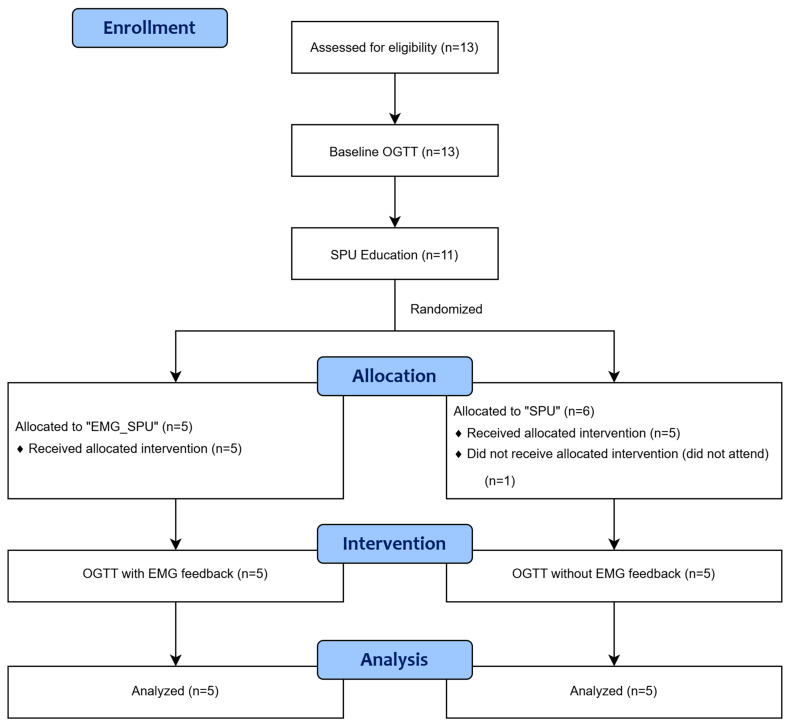
Study design.

The data collection and interventions for the study were conducted at the Musculoskeletal Rehabilitation Institute of the Szent György University Teaching Hospital of Fejér County in Csákvár during the second half of 2023. The research was approved by the Institutional Research Ethics Committee of the Szent György University Teaching Hospital of Fejér County and the Director General. The study was also approved by the National Public Health Center of Hungary (reference number 26914-5/2021/EÜIG). The flowchart of the study design is shown in Figure 1.

### 2.3. Interventions

#### 2.3.1. OGTT

The OGTT was conducted in the morning after a minimum of 10 h of fasting. For the three days preceding the test, participants adhered to a diet containing at least 150 g of carbohydrates per day while maintaining an average level of physical activity. Prior to initiating the test, fasting blood glucose levels were measured. If the value was below 7 mmol/L, the study proceeded. Following the initial (0 min) blood glucose measurement, participants consumed 75 g of glucose (Magilab Ltd., 1061 Budapest, Király utca 12, CAS number: 50-99-7) dissolved in 250–350 mL of water within five minutes. Blood samples were collected at nine time points, at 15 min intervals, over the subsequent 120 min. Capillary blood samples were obtained from a warmed hand via fingertip prick whenever feasible. The validity and reliability of this method for blood glucose measurement have been internationally recognized [8,9]. Blood glucose levels were assessed using an Accu-Chek Active device (Roche Hungary Ltd., Budapest, Hungary) and compatible test strips, both of which comply with the International Organization for Standardization (ISO) 15197:2013 standards [10].

In the group performing SPU without EMG feedback, the 105 min and 120 min blood glucose measurements for one participant were not recorded due to circumstances beyond our control.

#### 2.3.2. SPU Education

During the SPU education session, participants were instructed on the proper and reproducible execution of the SPU. A wireless EMG electrode (FREEMG 1000, BTS S.p.A., Milan, Italy) was placed on the soleus muscle venter of the preferred lower limb, following SENIAM recommendations [11].

**Figure 2:**
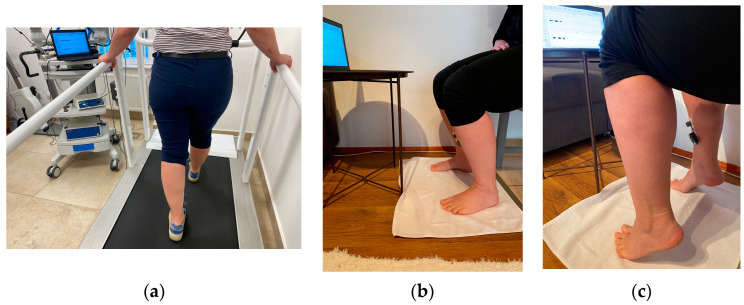
(**a**) Participants walked on a treadmill at a comfortable pace while receiving realtime EMG feedback on laptop monitor. (**b**) After a few minutes of walking, participants transitioned to a seated position with the knee bent in order to (**c**) perform the soleus push-up.

Participants first walked on a treadmill at a comfortable speed while receiving real-time EMG feedback on a laptop monitor (Figure 2a). The raw EMG signals, recorded at 1000 Hz, were rectified, smoothed, and displayed on the screen. After a few minutes of walking, participants transitioned to a seated position with the knee bent so that the metatarsophalangeal joint line fell below it, minimizing gastrocnemius muscle activity (Figure 2b) [5,12].

Participants then performed SPUs (Figure 2c), executing plantarflexion movements within an approximate 0–45° range at a cadence of ~60 repetitions per minute. The goal was to sustain markedly higher soleus muscle activity than during treadmill walking. All participants successfully learned the movement within a single session without reporting any adverse events.

#### 2.3.3. OGTT with SPU Intervention

One day after the SPU education session, participants underwent a second oral glucose tolerance test (OGTT) while performing SPUs. Depending on their assigned group, participants either received real-time EMG feedback (“EMG_SPU group”) or performed SPUs without feedback, estimating the intensity based on prior practice (“SPU group”).

In both groups, participants followed the same pre-test protocol as during the baseline OGTT, including a minimum 10 h of fasting before testing and a diet containing at least 150 g of carbohydrates per day for the preceding three days. Fasting blood glucose levels were measured prior to initiating the test, and if the value was below 7 mmol/L, the OGTT proceeded. After the 0 min blood glucose measurement, participants consumed 75 g of glucose dissolved in 250–350 mL of water within five minutes.

During the subsequent 120 min OGTT, participants continuously performed SPUs. In the “EMG SPU group”, real-time EMG feedback on soleus muscle activation was displayed on a laptop monitor, similar to the SPU education session. In the “SPU group”, participants performed SPUs without feedback, estimating intensity based on prior experience. Blood glucose levels were measured every 15 min, as in previous tests. Participants were instructed to avoid any additional physical activity during the OGTT. One participant’s blood glucose measurement was not recorded from the 75th minute due to technical issues. We requested that participants report any discomfort, fatigue, or muscle cramps immediately. No adverse events were reported.

### 2.4. Statistical Analysis

For statistical data analysis, Microsoft Excel (Microsoft Corporation, 2402 build version 16.0.17328.20124, 64-bit) was used for data recording and basic mathematical operations. To determine the incremental area under the curve (iAUC) defined by blood glucose levels measured every 15 min during the oral glucose tolerance tests (OGTT), as well as to assess the statistical significance of changes in blood glucose levels at specific measurement time points within and between groups, a mixed effects model with Tukey’s multiple comparison test at a 95% confidence level was applied. The analysis was conducted using GraphPad Prism 10 (GraphPad Software, Boston, MA, USA). To examine differences in iAUC within and between groups, we employed a one-factor analysis of variance at a 95% confidence level using IBM SPSS Statistics for Windows, Version 26.0 (IBM Corp., Armonk, NY, USA).

Normality was assessed using the Shapiro–Wilk test, and QQ plots were examined for further confirmation. As normality was observed across all groups, parametric methods were deemed appropriate. The mixed effects model was chosen for its ability to account for repeated measures and correct for missing values without reducing statistical power. Statistical tests were two-sided, and significance was set at *p* < 0.05.

## 3. Results

### 3.1. Characteristics of the Participants

The participants (n = 10) consisted of six male and four female volunteers, with an average age of 53.3 ± 2.7 years.

### 3.2. Performing SPU Results in Improved Postprandial Glucose Tolerance

**Figure 3:**
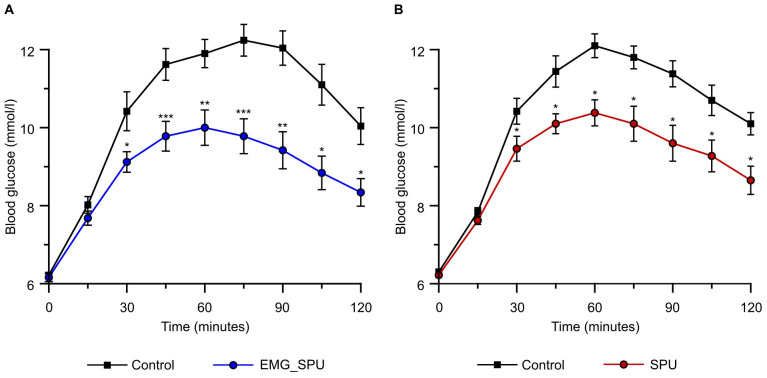
Performing soleus-dominant exercise is an effective way of immediately achieving improved glucose tolerance. Comparing the effect of SPUs on the group blood glucose level during the 120 min glucose tolerance test. (**A**) Performing soleus push-ups while continuously receiving EMG feedback about the soleus muscle activity (EMG_SPU) versus control measurement (Control) (n = 5). (**B**) Performing soleus push-ups while not receiving EMG feedback (SPU) versus control measurement (Control) (n = 5). Mean ± SEM. * *p* < 0.05, ** *p* < 0.005, *** *p* < 0.001 versus control measurement. Mixed effect model followed by Tukey’s multiple comparison test was used to evaluate the effect of SPUs. See Table 1 for detailed comparison at each time point.

**Figure 4:**
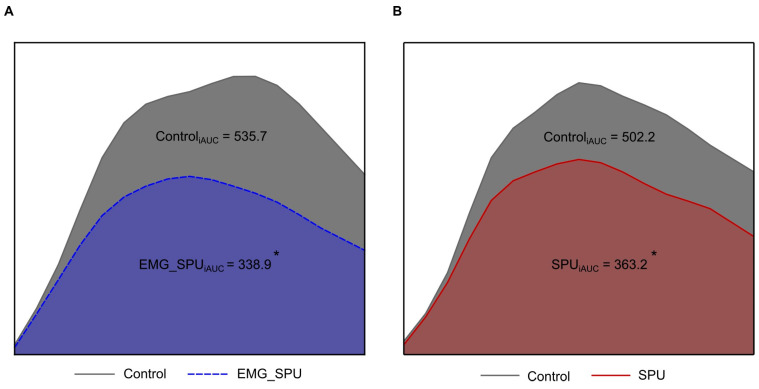
Repetitive soleus activity decreases the blood glucose incremental area under the curve by ~32%. Data represented as incremental area under the curve (iAUC), calculated by using the trapezoid method. Colored areas show the blood glucose iAUC change (**A**) in the group using continuous EMG feedback (EMG_SPU) versus control (n = 5) and (**B**) in the group without EMG feedback (SPU) versus control (n = 4). Mixed effect model, followed by Tukey’s multiple comparison test, was used to evaluate the difference of the iAUC between the interventional and the control. * denotes significance.

The effect of performing SPU on blood glucose levels during the two-hour-long glucose tolerance test is described in Table 1. No significant difference was observed between the group means for fasting blood glucose levels. A comparison of the means of the blood glucose values measured during the two-hour oral glucose tolerance test (OGTT) during the intervention, compared to those of the control OGTT, revealed a significant difference from the third sampling time onward and lasted until the last measurement time. Significant reductions in blood glucose levels were observed in both groups compared to the control. The graphical design of Table 1 and Figure 3 and Figure 4 was inspired by Hamilton’s study, with the aim of a better comparison of the results.

The effect of SPU was observed in both groups from the 30th minute of the oral glucose tolerance test until the final measurement time point. During the period of the OGTT, the blood glucose levels of the group that performed the intervention with EMG feedback were, on average, 2 mmol/L lower than those of the control measurement. In comparison, the group that performed SPU without EMG feedback exhibited a mean reduction of 1.49 mmol/L during SPU compared to the control measurement. A visual comparison of the means of blood glucose values during the intervention versus control measurements for the EMG feedback-guided group is shown in Figure 2a, and a comparison of the means of blood glucose values during the intervention versus control measurements for the group that did not receive continuous EMG feedback is shown in Figure 3B.

The trapezoidal rule was employed to ascertain the incremental area under the curve (iAUC), defined by the mean of the blood glucose values during the control oral glucose tolerance test and the mean of the blood glucose values measured during the oral glucose tolerance test during SPU, for both the group performing SPU with EMG feedback and the group performing SPU without EMG feedback. The difference in the iAUC of the control OGTT and the intervention OGTT for the group performing SPU with EMG feedback is illustrated in Figure 3A, while the corresponding figure for the group performing SPU without EMG feedback is Figure 3B. The repetitive soleus contractions resulted in a 37% reduction in glucose iAUC for the group performing the SPUs with EMG feedback. A −26% change was observed in the group that did not use external feedback during the SPUs.

**Figure 5:**
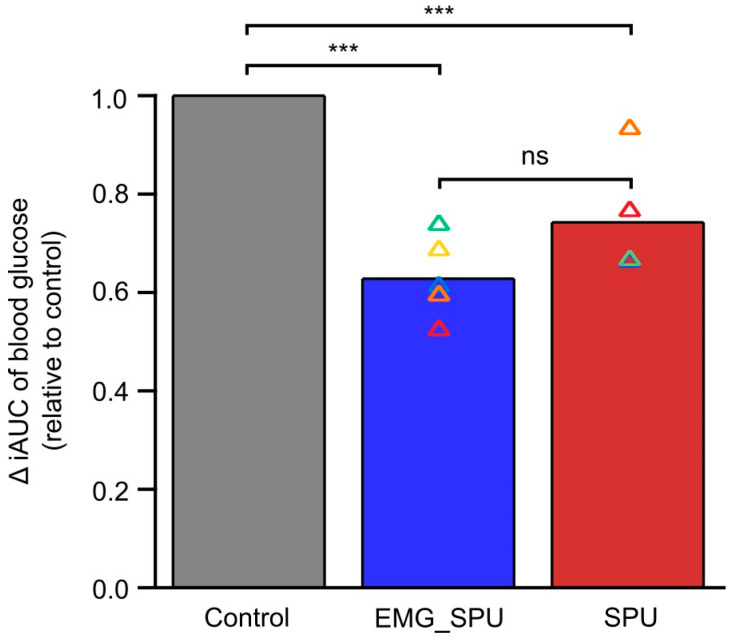
Prolonged soleus muscle activity improves glucose tolerance with and without electromyographic feedback. Colored triangles represent each individual glucose iAUC when performing soleus push-ups relative to the control measurement. Bar charts summarize the mean change in the group iAUC relative to the control results in the group that used EMG feedback (EMG_SPU) and in the group that did not use external biofeedback while performing the SPUs (SPU). Mixed effect model, followed by Tukey’s multiple comparison test, was used to evaluate the difference of the iAUC between the interventional and the control measurement. *** *p* < 0.001 versus control. ns = *p* > 0.05.

It was observed in both groups that performing prolonged soleus muscle activity significantly reduced the blood glucose iAUC compared to the control results. The smallest iAUC reduction, at 10%, was observed in the group not using EMG feedback during the contractile activity, while an individual in the group that used continuous EMG feedback achieved a 47% reduction. No significant difference was observed in the extent of reduction in the area under the curve between the two groups (Figure 5).

## 4. Discussion

The objective of our pilot study was to ascertain the impact of SPU exercise on glucose regulation in a small group of individuals with prediabetes. Additionally, we sought to examine the feasibility of integrating SPU into daily life, given that continuous EMG feedback was not available for one group.

The research of Hamilton and colleagues [5] formed the basis of our research; however, it differed to some extent. Hamilton and colleagues carried out two separate experiments. In Experiment II, 15 individuals completed a 3-hour-long OGTT while sedentary and during at least one activity condition. All 15 subjects completed a 3-hour-long OGTT while performing SPUs at 1.3 metabolic equivalents [METs, 1 MET = 3.5 mL oxygen/kg/min], and 10 of the 15 individuals were also tested at a 1.7 MET activity level. This randomized counterbalanced design, along with the sedentary control testing, was set up in order to test if the intensity of soleus contraction would impact glucose and insulin reductions. While both activity levels of SPUs resulted in a significantly decreased blood glucose iAUC compared to the sedentary results, performing SPUs at 1.7 METs led to a more robust decrease compared to performing SPUs at 1.3 METs.

Beforehand, Hamilton and colleagues carried out another experiment, Experiment I, which involved different sedentary subjects (n = 10). The goal was to investigate fatigue and glycogen use during SPUs at an even higher activity level (2 METs) for 270 min. They found that the glycogen stored in the soleus muscles contributed minimally to the prolonged local muscle activity at both 130 and 270 min while both fat oxidation and total carbohydrate oxidation increased to fuel soleus muscle contractions. Participants did not report any cramps, joint or muscle pain, and no adverse events took place during the 270-minute-long SPU session [5].

In our pilot study, the 10 participants underwent a 2-hour-long OGTT while sedentary, serving as the control results. Later, each subject repeated the OGTT while performing SPUs, either with or without continuous EMG feedback on the intensity of the SPUs. In this pilot study, the METs were not determined in either of the two groups. We aim to repeat the study with an increased sample size at more centers in the future.

It is notable that Hamilton and colleagues investigated the efficacy of SPU in healthy individuals, whereas our study focused on examining the impact of SPUs in individuals with prediabetes. It is important to highlight that although having an IFG was an exclusion criterion in the Hamilton 2022 study, the subjects were in the IGT range at the 120th minute during the sedentary OGTT [5].

As with the study conducted by Hamilton et al., our findings indicated that SPU had a beneficial impact on glucose tolerance between minutes 30 and 120 of the oral glucose tolerance test, resulting in a one-third reduction in the area under the curve of blood glucose values during the OGTT. Furthermore, the beneficial impact of SPU was evident even in the absence of EMG feedback.

In support of the mechanistic discussion of the metabolic background of SPU by Hamilton et al. [5], the current authors propose a novel theory regarding the relevance of the activation of the unaccounted Piezo system in first-line insulin regulation. Accordingly, we theorize that the soleus muscle is not solely a muscle but rather a proprioceptive organ with a high muscle spindle content. Proprioception is the awareness of the position and movement of limbs. This hypothesis is supported by earlier animal studies [13]. This function of the soleus muscle may be of even greater importance in humans, given the transition from a quadrupedal to a bipedal stance, which has resulted in a higher need for the contribution of Piezo2 ion channels to proprioception [14]. It is noteworthy that Ardem Patapoutian and his team demonstrated that Piezo2 is the principal mechanosensitive ion channel involved in proprioception [15]. Moreover, innocuous stimuli that induce mechanical allodynia are often part of diabetes-associated nerve damage, and the involvement of mechanosensitive Piezo2 has been implicated in this mechanism [16]. Furthermore, the inhibition of the Piezo2 pathway blocks the development of mechanical allodynia [17].

Mechanotransduction is hierarchical [18], and it is probable that Piezo2 is the initial activating mechanism in proprioceptive terminals at the onset of exercise due to its ability to induce a burst of activation [19]. It is also important to consider the possibility of crosstalk between somatosensory Piezo2 and peripheral Piezo1 [20,21]. Piezo1 has been demonstrated to possess mechanosensory properties in capillaries, and it is postulated that it may play a regulatory role in the distribution of local blood flow through the nervous system [22]. Furthermore, Piezo1 channels are capable of sensing and responding not only in a spatially restricted manner [23] but also at the whole-body level [24]. This enables them to enhance performance and reset homeostasis, which is regulated by the nervous system through the aforementioned Piezo2–Piezo1 crosstalk [25].

The latest research has revealed that Piezo2 may induce an intrinsic oscillatory interoceptive mechanism via pressure pulse transduction detection, which alters olfactory bulb activity during arousal and is synchronized with brain activities [26]. The hypothesis of long-range synchronization initiated by Piezo2 was previously postulated in the context of the brain [27] and proposed as a potential mechanism from the periphery, specifically from the muscle spindle to the brain [28]. These new research findings demonstrate the importance of Piezo2 in arterial pressure pulse detection and underpin an ultrafast oscillatory synchronization mechanism that may be impaired in the muscle spindle during delayed onset muscle soreness (DOMS) instigated mechanical hyperalgesia. It is noteworthy that mechanical hyperalgesia is also often induced in diabetes [29].

The relationship between DOMS and amyotrophic lateral sclerosis (ALS) pathomechanism theory is discussed in the aforementioned paper [28]. Accordingly, the transient or irreversible microdamage of Piezo2 in intrafusal proprioceptive terminals is postulated to be the “gateway to pathophysiology” in both DOMS and ALS [28]. The prominent role of the soleus is also reflected in the induction of the so-called “gateway reflex”, which has the potential to be elicited in other spinal segments, not only in the soleus-innervating L5 segment [30]. It is proposed that the acute transient form of this reflex is the Piezo2 channelopathy-induced inflammatory reflex [31], a neural circuit-based defense system that maintains neuroimmune homeostasis. In contrast, the chronic form is the aforementioned “gateway reflex”, which involves the “gateway to pathophysiology” in its initial stages [31].

It can therefore be hypothesized that this Piezo2 channelopathy induces impairment or loss of long-range proton-based ultrafast synchronization to the hippocampus, both temporarily in the case of DOMS and permanently in ALS [28]. Moreover, this theory of ultrafast proton-based long-range neurotransmission has also been proposed to play a role in first-line insulin regulation [28]. It is also worth noting that this proton-based ultrafast signaling is implicated in this process, with the involvement of catalytically inactive carbonic anhydrase (CA) acting as proton-collecting and -distributing antennas, as demonstrated in proprioceptive sensory neurons and motoneurons [28]. It is noteworthy that animal research indicates that during puberty, the soleus muscle undergoes a transformation from fast-twitch oxidative-glycolytic to slow-twitch oxidative fibers, accompanied by a shift in the status of CA from sensitive to resistant form [32]. The resistant isoform of CA, CA III, is present in the soleus muscle, axons, intrafusal fibers, and capillary endothelial cells [32]. This transformation, as postulated by the current authors, is thought to play a role in proton-based ultrafast proprioceptive signaling, given that it acts as a proton-collecting and -distributing antenna. It is also noteworthy that the soleus muscle is a key player in supporting proprioception, postural control, and bipedality.

It seems reasonable to suggest that SPU may be an effective exercise method for initiating the Piezo system in sedentary individuals by promoting proton-based long-range ultrafast synchronization. It is hypothesized that this ultrafast signaling is impaired in prediabetes. SPU may therefore serve as an exercise technique to promote these ultrafast proton-based synchronization pathways, thereby leading to more optimal instant or first-line insulin regulation in response to mechanotransduction.

Furthermore, unaccustomed or strenuous eccentric contractions have been demonstrated to cause DOMS, along with associated neuromuscular changes that persist for several days [33,34,35]. Additionally, the severity of eccentric exercise-induced muscle damage has been shown to correlate with the degree of insulin resistance [36]. It is important to note that DOMS is proposed to be a biphasic non-contact injury mechanism, in which the primary damage is suggested to be an acquired Piezo2 channelopathy at the proprioceptive terminals of the muscle spindle [25]. Sonkodi et al. attributed the negative correlation to an atypical hippocampal-like metabotropic phospholipase D (PLD)-mGluR on proprioceptive primary afferents, which were first identified by Thomson et al. [37] and are homomeric to metabotropic GluK2 [38]. Indeed, Gluk2 plays a role in the regulation of glucose homeostasis [39]. However, this negative homeostatic regulation is hypothesized to be disrupted by the Piezo2 channelopathy-induced DOMS effect. Consequently, the impaired Piezo2–Piezo1 crosstalk resulting from acquired Piezo2 channelopathy may lead to the abrogation of proper Piezo1 modulation, which could, in turn, result in insulin resistance, given that Piezo1 is involved in glucose-induced insulin secretion [40]. In support, a recent pilot study demonstrated that a DOMS-inducing exercise protocol impaired orthostasis in a similar fashion to that observed in diabetes [38].

In conclusion, our SPU protocol, which is similar to the one described by Hamilton et al. [5], did not result in the induction of DOMS due to the enigmatic metabolic and fatigue resistance characteristics of the soleus muscle. Moreover, this exercise regimen appears to facilitate the unidentified novel Piezo2-mediated ultrafast proton-based synchronization pathways, thereby promoting more optimal insulin regulation in response to mechanotransduction, even in individuals with prediabetes. Therefore, SPU exercise seems to offer multiple physiological benefits, including improved blood glucose regulation, enhanced circulation, increased energy production, and optimized metabolic processes. These effects are particularly relevant for individuals with prediabetes, insulin resistance, or those with sedentary lifestyles. Due to its low-intensity nature, SPU is easily integrated into daily routines and serves as a sustainable long-term intervention.

## 5. Limitations of the Pilot Study

As this is a pilot study, one of its inherent limitations is the small sample size; therefore, a larger sample size is warranted for validation. The primary goal of pilot studies is to assess feasibility and refine methodologies, and as such, a larger sample size would be necessary to draw more definitive conclusions. Additionally, the self-reporting of dietary intake introduces the potential for bias, as participants may have under- or over-reported their food consumption. This could have affected the accuracy of the OGTT results, as precise dietary control is crucial for obtaining reliable glucose metabolism measurements.

Additionally, while we confirmed normality in our dataset using the Shapiro–Wilk test and QQ plots, it is important to acknowledge that normality assessment with a small sample size has inherent limitations. Although parametric methods were justified and the mixed effects model allowed for the correction of missing values, future studies with larger samples would provide a more robust statistical foundation.

Future studies with larger sample sizes and more objective dietary monitoring are warranted to further validate the reported findings.

## 6. Conclusions

The findings of our unique exercise protocol-driven pilot study for individuals with prediabetes suggest that SPU can be performed for at least two hours without inducing fatigue, while also improving postprandial glucose tolerance. Therefore, SPU is suggested to be a cost-effective, accessible, and equipment-free exercise with systemic metabolic benefits. However, further validation in larger cohorts is required to confirm its efficacy.

## Figures and Tables

**Table 1:** Blood glucose levels at each measurement point.

OGTT	With EMG Feedback			Without EMG Feedback		
Time (Minutes)	Control (n = 5)	SPU (n = 5)	Δmmol/L	Control (n = 5)	SPU (n = 5)	Δmmol/L
**0**	6.2 ± 0.1	6.2 ± 0.1	−0.1 ± 0.1	6.3 ± 0.1	6.2 ± 0.1	−0.1 ± 0.1
**15**	8 ± 0.2	7.7 ± 0.2	−0.3 ± 0.2	7.8 ± 0.1	7.6 ± 0.1	−0.2 ± 0.1
**30**	10.4 ± 0.5	9.1 ± 0.3	−1.3 ± 0.3 *	10.4 ± 0.3	9.5 ± 0.3	−1 ± 0.2 *
**45**	11.6 ± 0.4	9.8 ± 0.4	−1.8 ± 0.2 ***	11.4 ± 0.4	10.1 ± 0.3	−1.3 ± 0.3 *
**60**	11.9 ± 0.4	10 ± 0.5	−1.8 ± 0.1 **	12.1 ± 0.3	10.4 ± 0.3	−1.7 ± 0.3 *
**75**	12.2 ± 0.4	9.8 ± 0.4	−2.5 ± 0.2 ***	11.8 ± 0.3	10.1 ± 0.4	−1.7 ± 0.4 *
**90**	12 ± 0.4	9.4 ± 0.5	−2.6 ± 0.3 **	11.4 ± 0.3	9.6 ± 0.5	−1.8 ± 0.3 *
**105**	11.1 ± 0.5	8.8 ± 0.4	−2.3 ± 0.4 *	10.7 ± 0.4	9.3 ± 0.4	−1.4 ± 0.4 *
**120**	10 ± 0.5	8.3 ± 0.4	−1.7 ± 0.4 *	10.1 ± 0.3	8.7 ± 0.4	−1.5 ± 0.3 *

Mean ± SEM. Mixed effect model, followed by Tukey’s multiple comparison test, was used to evaluate the effect of soleus push-up (SPU) on blood glucose values during the oral glucose tolerance test (OGTT). * *p* < 0.05, ** *p* < 0.005, *** *p* < 0.001 versus control measurement. The difference between SPU and control is represented at each time point (Δmmol/L).

## Data Availability

The data presented in this study are available upon request from the corresponding author.

## Abbreviations

OGTT	Oral Glucose Tolerance Test
SPU	Soleus push-up
EMG	Electromyograph
OECD	Organization for Economic Co-operation and Development
IFG	Impaired fasting glucose
IGT	Impaired glucose tolerance
iAUC	Incremental area under the curve
